# Fecundability and Sterility by Age: Estimates Using Time to Pregnancy Data of Japanese Couples Trying to Conceive Their First Child with and without Fertility Treatment

**DOI:** 10.3390/ijerph18105486

**Published:** 2021-05-20

**Authors:** Shoko Konishi, Fumiko Kariya, Kisuke Hamasaki, Lena Takayasu, Hisashi Ohtsuki

**Affiliations:** 1 Department of Human Ecology, School of International Health, Graduate School of Medicine, The University of Tokyo, Tokyo 113-0033, Japan; kariya@humeco.m.u-tokyo.ac.jp (F.K.); hamasaki@humeco.m.u-tokyo.ac.jp (K.H.); lena.takayasu@humeco.m.u-tokyo.ac.jp (L.T.); 2 Department of Anthropology, University of Washington, Seattle, WA 98195, USA; 3 Department of Evolutionary Studies of Biosystems, School of Advanced Sciences, The Graduate University for Advanced Studies, Kanagawa 240-0193, Japan; ohtsuki_hisashi@soken.ac.jp

**Keywords:** age, assisted reproductive technology, beta distribution, fecundity, fertility, maximum likelihood, time to pregnancy

## Abstract

Fecundability, the probability of conception in a month or in a menstrual cycle, varies across and within age groups for both women and men. Fertility treatment has become common in a number of countries including Japan, but its impact on the age pattern of fecundability is unknown. By utilizing the previously collected data on time to pregnancy (TTP) of Japanese couples trying to conceive their first child, the present study aimed to estimate fecundability and sterility by women’s age and to assess how the estimates may differ by including or excluding assisted conceptions. Duration between discontinuing contraception and conception (including both natural and assisted) resulted in a live birth was called TTP-all, and the duration ending with natural conception was called TTP-natural. TTP-natural was censored when a participant received fertility consultation or treatment. A zero-inflated beta distribution model was used to estimate a proportion of sterile (zero probability of conception) and a distribution of fecundability for each age group. Parameters of the distribution were estimated using the maximum likelihood method. When TTP-all and TTP-natural were used, the sterile proportion of the whole sample was, respectively, 2% and 14%, and the median (interquartile range) of fecundability was, respectively, 0.10 (0.04, 0.19) and 0.11 (0.05, 0.19). The median (interquartile range) of fecundability was 0.18 (0.10, 0.29) for women aged 24 years or younger and 0.05 (0.02, 0.13) for 35–39 years old when TTP-all was used, and the estimates were quite similar with those based on TTP-natural: it was 0.18 (0.10, 0.29) for women aged 24 years or younger and 0.06 (0.00, 0.15) for 35–39 years old. Exclusion of assisted conceptions resulted in larger proportions of sterility, but it had little impact on median or interquartile ranges of fecundability estimates. Fecundability is overall lower at higher ages, while interquartile ranges are overlapping, suggesting that inter-individual variability of fecundability within an age group is as large as the variability across age groups.

## 1. Introduction

Age-related changes in fertility and fecundity, the biological ability to reproduce, have been an interest to demographers, clinical doctors, and those trying to conceive their child. Age-specific marital fertility rates of natural fertility populations showed a common age pattern for women: it is highest for the ages 20–24 years and declines at higher ages, while the absolute values of fertility varied across populations [1] (p. 22), [2] (p. 54). The age-specific marital fertility observed in natural fertility populations are believed to reflect the age pattern of fecundity. Fecundity can be lowered due to various factors including ovulatory disorders, a lower frequency of sexual intercourse, a lower probability of conception from a single insemination, and a higher probability of fetal loss [2] (p. 71).

While fecundity cannot be directly measured, fecundability, the probability of conception in a menstrual cycle [3] or in a month, can be calculated from observed data. Using the data on birth intervals from two natural fertility populations, Bendel et al. [4] estimated the age pattern of fecundability and found that decline in fecundability after 25 years old was more pronounced compared to the decline in fertility. A classic study [5] that targeted nulliparous women who had azoospermic husbands and received artificial insemination found that fecundability is lower for women aged 30 or higher, and the age effect was more pronounced after 35 years of age. More recently, time to pregnancy (TTP), number of months or cycles at risk of pregnancy, i.e., having unprotected intercourse, before conception, has been used to estimate the effect of age and other factors on fecundability. For example, a study [6] that followed nulliparous and parous women for up to 12 months found that fecundability was highest for women at around 30 years of age. Konishi et al. [7] targeted nulliparous Japanese women and reported that fecundability was lower for women aged 27 or higher, compared to those aged 24–26 years. The age patterns of fecundability reported in the above-mentioned studies are slightly inconsistent, partly because the definitions of fecundability and target populations as well as the statistical methods used varied across studies. Wood [2] differentiated three types of fecundability. Effective fecundability is calculated with only conceptions resulted in a live birth, apparent fecundability is calculated with all detected conceptions regardless of the pregnancy outcome, and total fecundability can theoretically be calculated using all (including both detected and undetected) conceptions. In the present analysis, we use the term fecundability to refer to effective or apparent fecundability.

It is empirically known that even with the same age fecundability varies across individuals [2]. Weinberg and Gladen [8] used the beta distribution to model the distribution of fecundability and potential impacts of covariates. Wood et al. [9] constructed a multistate hazards model to simultaneously estimate fecundability and sterility (zero fecundability), and transition from fecund (non-zero fecundability) to sterile state. Dunson et al. [10] constructed a Bayesian model to estimate fecundability using daily data on ovulation and sexual intercourse. Several other studies modeled heterogeneity in fecundability [11]. These models are able to incorporate both a fraction of sterility and heterogenous fecundability within a subset of fecund individuals.

During the past several decades fertility treatment has become common worldwide [12]. While such medical intervention can help couples have a baby who would otherwise not be able to conceive naturally, it is unknown how it may affect age pattern of fecundability and sterility at a population level. In previous studies that estimated fecundability using TTP, couples were censored when they initiated fertility treatment [6,13]. Considering that an increasing proportion of births are from assisted conceptions, censoring TTPs at fertility treatment may impact the estimated age pattern of fecundability, but it is not known how it may or may not affect estimates. Thus, the question we would like to address in the current paper is how fertility treatment can affect fecundability distribution and its age pattern at a population level. To address this question, we utilized the data from our previous study on age and TTP of Japanese couples trying to conceive their first child [7]. In the study, 22% of the analytic sample had ever received fertility consultation or treatment before the first birth [7].

By utilizing the previously collected data on TTP of Japanese couples trying to conceive their first child, the present paper aimed to estimate fecundability and sterility by women’s age and to assess how the estimates may differ by including or excluding assisted conceptions.

## 2. Materials and Methods

### 2.1. Data

In the present paper, we defined TTP in two ways. Duration between discontinuing contraception and (either natural or assisted) conception ended in a live birth or an ongoing pregnancy was called TTP-all, whereas the duration ending with natural conception ending in a live birth or an ongoing pregnancy was called TTP-natural. TTP-natural was censored when a participant received fertility consultation or treatment or when a participant had not given birth nor currently pregnant at the time of survey. TTP-all was only censored when a participant had not given first birth and was not currently pregnant at the time of survey.

We used the secondary data from a study on TTP targeting Japanese couples trying to conceive their first child, as well as their data on the timing of initiating fertility consultation or treatment [7]. To exclude the potential recall bias, TTP was truncated at 60 months. In other words, only those who discontinued contraception within the past 60 months from the survey to conceive their first child was included in the analysis. Detailed description about the analytic sample can be found in [7]. From the analytic sample (n = 1324) used in the previous paper [7], 60 samples were further excluded because the data on the duration between discontinuing contraception and initiation of fertility consultation or treatment were unknown. The analytic sample of the present study (n = 1264) consists of three groups: parous women (Group A, n = 787), nulliparous and currently pregnant women (Group B, n = 165), and nulliparous and non-pregnant women who were at risk of pregnancy, i.e., having an unprotected intercourse, at the time of survey (Group C, n = 312).

TTP-all was non-censored, i.e., the duration between discontinuing contraception and conception was known, for all women of Groups A and B, whereas it was censored for all women of Group C. TTP-natural was either non-censored or censored for women of Groups A and B, whereas it was censored for all women of Group C. Fecundability is affected by both female and male factors and also their combinations, but in this paper we simply refer to women, because the focus of the paper is the distribution of fecundability and sterility by women’s age.

### 2.2. Statistical Analyses

All statistical analyses were conducted using Matlab R 2020a. When a *p*-value was lower than 0.01, it was considered a statistically significant result.

We used three types of models (with one, two, and three parameters) to describe the distribution of fecundability of the study population. The one-parameter model assumed that fecundability is constant across observation time (up to 60 months) and the same for individuals in the same age group (homogeneous fecundability). A common assumption applied to the two- and three-parameter models was that fecundability *P* is constant for all women across observation time (up to 60 months) and *P* varies across individuals (heterogeneous fecundability). The two-parameter model assumed that the fecundability *P* follows a beta distribution within each age group [8]. The three-parameter model assumed that a fraction *q* of women has an absolutely zero probability of conception (=sterile) and the rest of women’s fecundability follow a beta distribution within each age group [8]. The latter model is also called a zero-inflated beta distribution model.

### 2.3. Maximum Likelihood Method

The one-, two-, and three-parameter models were constructed for each age group (24 or younger, 25–29, 30–34, 35–39, and 40+ years old) and each type of TTP (TTP-natural and TTP-all), and parameters were estimated using the maximum likelihood method (Appendix A). The age groups are based on ages at discontinuing contraception (=start of TTP). These age groups were used because they are generally used in demography and fertility statistics.

Maximum log-likelihood was calculated using the estimated parameters of each model. To test if heterogenous fecundability (compared to homogeneous fecundability) fits better with the observed TTPs (all or natural), a likelihood ratio test was conducted using the maximum log-likelihood of one- and two-parameter models. Similarly, to test if the assumption of a sterile fraction fits the observed TTPs better, a likelihood ratio test was conducted using the maximum log-likelihood estimates of the two- and three-parameter models.

With the estimated parameters (alpha and beta) of the two-parameter models, means and variance of fecundability were calculated. In addition, the median and the first and third quartiles of fecundability were calculated using the estimated distributions of the two- and three-parameter models.

### 2.4. Sensitivity Analysis

Parameters of the two-parameter models were estimated using the TTP-natural truncated at 24 months (n = 525) to examine how truncation can affect the estimated distribution of fecundability and sterility by age.

## 3. Results

Among the analytic sample of 1264 women, 952 conceived by the time of survey (Table 1). The mean (SD) of TTP-all, including both censored and non-censored, was 10.3 (12.9) months for all ages, and it tended to be longer for women of higher ages (Table 1). A total of 834 women conceived without receiving fertility consultation or treatment, and the mean (SD) of TTP-natural, including both censored and non-censored, was 8.2 (10.4) months. The SDs of both TTP-all and TTP-natural were as large as the means (Table 1). A total of 230 women received fertility consultation or treatment before conceiving their first child. The numbers and proportions of women who received fertility consultation or treatment by age at discontinuing contraception were 5 (5%), 69 (12%), 95 (23%), 50 (29%), and 11 (52%) for 24 or younger, 25–29, 30–34, 35–39, 40+ years old, respectively (Table S1).

A likelihood ratio test showed that compared to the one-parameter models the two-parameter models fits better with the observed TTP-all and TTP-natural for the all age categories except for 40+ years (Table A1). On the other hand, the differences between the two- and three-parameter models was not statistically significant except for all ages with TTP-natural (Table A2).

With the two-parameter model, estimated alpha was 0.94 and beta was 6.22 for all ages using TTP-all, and alpha was 0.84 and beta was 5.44 when TTP-natural was used (Table 2). The median (interquartile range) of fecundability was 0.10 (0.04, 0.19), and it was the same for both TTP-all and TTP-natural. The median fecundability was 0.18 and highest for women aged 24 or younger, and it tends to be lower with advanced age, which trend was again the same for both types of TTP (Table 2).

With the three-parameter model, estimated parameters clearly differed between TTP-all and TTP-natural (Table 3). The sterile proportions for all ages were 2% with estimates based on TTP-all, while it was 14% with TTP-natural. When TTP-all was used, the proportion of sterility by age group ranged between 0% and 3%, whereas it ranged between 0% and 30% when TTP-natural was used (Table 3). On the other hand, the median and the first and third quartiles of fecundability were similar regardless of the TTP type (Table 3). The median fecundability based on TTP-all was 0.18, 0.12, 0.09, 0.05, and 0.00 for women aged 24 or younger, 25–29, 30–34, 35–39, and 40+, respectively, and the interquartile ranges were overlapping across the age groups for 39 years or younger (Table 3).

**Figure 1:**
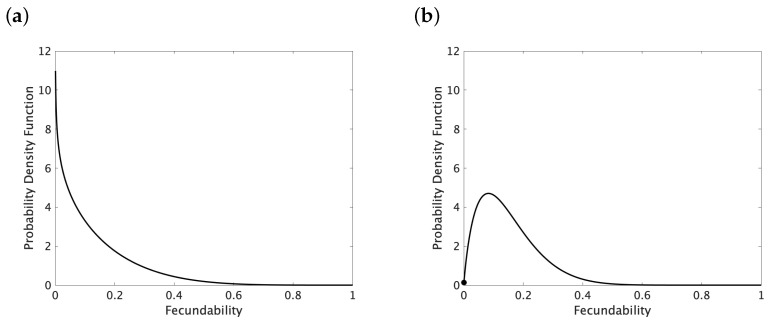
Probability density function of fecundability for all ages, based on: (**a**) two-parameter (α=0.84,β=5.44); or (**b**) three-parameter models (α=1.88,β=10.73,q=0.14). Estimates were made using TTP-natural (n = 1264). In (**b**), the probability mass of q=0.14 exists at zero-fecundability.

The probability density function of fecundability for all ages based on TTP-natural with either two- or three-parameter models are shown in Figure 1. With the two-parameter model that assumed no proportion of sterile couples, the probability density inflated to infinity at zero fecundability (Figure 1a), whereas, with the three-parameter model assuming a proportion of sterile women, the probability density did not inflate to infinity at zero (Figure 1b). Fecundability estimates remained overall similar in the sensitivity analysis when the analytic sample was truncated at 24 months (Table A3).

## 4. Discussion

When TTP-natural was used, sterile proportions were higher compared to when TTP-all was used. However, the estimated medians and interquartile ranges of fecundability were similar regardless of the type of TTP used. Fecundability was overall highest for women aged 24 years or younger and tended to be lower with advanced age, but the interquartile ranges were overlapping across age groups.

The age pattern of sterility differed significantly depending on how TTP was defined in the present study. When TTP was censored at the first fertility consultation or treatment (TTP-natural), the proportion sterile was as high as 30% for women aged 35 to 39 years old. On the other hand, when non-censored TTPs ending either with natural and assisted conceptions were included (TTP-all), the proportion of sterility was only 3% or lower for all age categories. The low proportion of sterility is consistent with the findings of previous studies [9,10]. Our findings suggest that censoring cycles at the first fertility consultation or treatment may result in overestimation of sterility. On the other hand, the means and interquartile ranges of fecundability were quite similar for estimates based on TTP-all and TTP-natural, which is probably linked to the fact that fecundability of those couples with relatively lower (but not with higher) fecundability could potentially be affected by such medical interventions.

The median fecundability was highest for women of 24 years old or younger, and lower towards the end of reproductive ages. This age pattern is similar to the findings of Dunson et al. [10], who utilized daily data on ovulation and sexual intercourse from couples in seven European centers. Woman’s age is related to the quality of oocytes, which is closely linked to fecundability [14,15]. According to our estimates based on TTP-all, proportions sterile do not vary across age groups; instead, fecundability tends to be lower in higher age groups. It indicates that overall longer TTP at higher ages can be attributed to a lower fecundability rather than to a larger proportion of sterile couples. A couple of previous studies [9,10], both assuming heterogenous fecundability, concluded that age-related increase in infertility is due to reduction in fecundability, rather than to an increase in sterility, which is consistent with our finding based on TTP-all. The age pattern of fecundability reported from studies using fecundability ratios (hazard ratios) derived from a survival analysis is slightly different from the current finding. A prospective cohort study of women trying to conceive [6] reported that there was little difference in fecundability for women aged 20–34, and women aged 35 or higher showed lower fecundability, after controlling for several volitional factors. When the samples were limited to nulliparous women, the age-related decline in fecundability was more pronounced [6]. The present sample consists solely of nulliparous women trying to conceive their first child, which can partly explain the apparently lower fecundability of older women. It is also important to note that in the present paper the analytic sample included only 21 women in the highest age group (40+ years). If more women of 40 years or older were included, age-related changes in fecundability may have been different. In addition, different assumption about fecundability can also explain the differences in age pattern observed in the present paper versus that in previous studies. With survival analysis, as employed in previous studies (e.g., [6,13]), fecundability of women in each sub-group is assumed to be homogenous, while in our analysis each woman was assumed to have heterogenous fecundability.

The present results show that variability of fecundability within each age group can be as large as the variability across age groups. The interquartile ranges of fecundability overlaps for age groups 24 or younger, 25–29, 30–34, and 35–39 years, which suggests that a 24-year-old at the 25th percentile of fecundability distribution can be less likely to conceive than a 39-year-old at the 75th percentile of the distribution. Factors other than age, including frequency of sexual intercourse, risk of fetal loss, probability of ovulation, probability of conception per insemination, lifestyle factors, and other unmeasured heterogeneity [6,16,17,18,19,20] can play a role. Elucidating impacts of volitional determinants of fecundability would be useful for those trying to conceive their child, because, unlike biological age, these factors can be modified by themselves. Efforts have been made to predict probability of giving birth using prediction models that targeted couples with unexplained subfertility (e.g., [21,22]). The present results showing heterogeneous fecundability within an age group supports the idea that considering not only a couple’s ages, but also other factors that can impact their fecundability is important to judge how long a couple should wait to conceive naturally and when they had better start fertility treatment. Factors that potentially affect a couple’s fecundability include exposures to endocrine disrupting chemicals such as pesticides [23], heavy metals [24], bisphenol A, and phthalates [25]. The mechanisms through which these environmental and lifestyle factors can affect couple’s fecundability need further investigation. A recent study reported potentially important roles of mitochondria in determining oocyte and embryo quality [26], which may also be linked to variability of fecundability across couples.

Limitations of the present study include that the fecundability model used did not allow each individual’s fecundability to vary across time, and that we do not know the true distribution of fecundability. The beta distribution has been used in previous studies because it has high flexibility with only two parameters. The analytic sample included those who planned their pregnancy and remembered TTPs, therefore we do not have information on fecundability of many others who did not plan their pregnancy. Nonetheless, to the best of our knowledge, the present study is the first to analyze the potential impact of censoring TTP with fertility consultation or treatment on the age pattern of fecundability and sterility. The present results also indicate that variability of fecundability within an age group can be as large as that across age groups. We hope that these findings would facilitate more research on fecundability and its correlates targeting general populations. Limitations of the present study include that the potential impact of socioeconomic status, reproductive history (e.g., fetal loss, stillbirth, and induced abortion), fertility treatment, and partnership status on fecundability were not examined due to the limited sample size. Age and other physiological characteristics (e.g., semen quality) and environmental exposures of their partner in relation to couple’s fecundability can also be a scope of future research.

## 5. Conclusions

Exclusion of assisted conceptions resulted in larger proportions of sterility, but it had little impact on median or interquartile ranges of fecundability estimates. Fecundability is overall lower at higher ages, while interquartile ranges are overlapping across age groups, suggesting that inter-individual variability of fecundability within an age group is as large as the variability across age.

## Figures and Tables

**Table 1:** Number of participants and all (including both assisted and natural) and natural conceptions resulted in a live birth or an ongoing pregnancy by ages at discontinuing contraception. The mean (SD) of TTP-all and TTP-natural by age at discontinuing contraception were calculated using both censored and non-censored TTPs.

Age (y) Group	Participants	All Conceptions	Natural Conceptions	TTP-All ${}^{a}$ (Month)	TTP-Natural ${}^{b}$ (Month)
All	1264	952	834	10.3 (12.9)	8.2 (10.4)
24 or younger	91	81	80	6.8 (9.7)	6.5 (9.0)
25–29	561	456	415	8.5 (11.2)	7.0 (9.2)
30–34	417	304	253	11.6 (14.0)	9.5 (11.9)
35–39	174	107	83	13.7 (15.0)	9.8 (10.3)
40+	21	4	3	19.7 (12.8)	8.9 (7.9)

${}^{a}$ TTP-all includes non-censored TTPs ending with either natural or assisted conceptions and censored TTPs ending at the time of survey without conception. ${}^{b}$ TTP-natural includes non-censored TTPs ending with natural conceptions and censored TTPs ending either at the time of survey or at the initiation of infertility consultation or treatment.

**Table 2:** Parameters of the two-parameter models and the median, the first and third quartiles, and the mean and variance of fecundability estimated using TTP-all and TTP-natural for all ages and by age group (n = 1264).

TTP-All								
		**Parameters**		**Fecundability**				
**Age (y) Group**	**n**	α	β	**First Quartile**	**Median**	**Third Quartile**	**Mean**	**Variance**
All ages	1264	0.94	6.22	0.04	0.10	0.19	0.13	0.01
24 or younger	91	1.21	4.48	0.09	0.18	0.31	0.21	0.03
25–29	561	1.44	8.76	0.06	0.12	0.20	0.14	0.01
30–34	417	0.82	5.91	0.03	0.09	0.18	0.12	0.01
35–39	174	0.61	5.98	0.02	0.05	0.13	0.09	0.01
40+	21	0.24	15.47	0.00	0.00	0.02	0.02	0.00
**TTP-Natural**								
		**Parameters**		**Fecundability**				
**Age (y) Group**	**n**	α	β	**First Quartile**	**Median**	**Third Quartile**	**Mean**	**Variance**
All ages	1264	0.84	5.44	0.04	0.10	0.19	0.13	0.02
24 or younger	91	1.24	4.60	0.09	0.18	0.30	0.21	0.02
25–29	561	1.46	8.87	0.06	0.12	0.20	0.14	0.01
30–34	417	0.58	3.90	0.02	0.08	0.19	0.13	0.02
35–39	174	0.42	3.87	0.01	0.04	0.14	0.10	0.02
40+	21	0.31	15.23	0.00	0.01	0.02	0.02	0.00

**Table 3:** Parameters of the three-parameter models and the median, the first and third quartiles of fecundability estimated using TTP-all and TTP-natural for all ages and by age group (n = 1264).

TTP-All							
		**Parameters**			**Fecundability**		
**Age (y) Group**	**n**	α	β	**q (Sterile)**	**First Quartile**	**Median**	**Third Quartile**
All ages	1264	1.01	6.60	0.02	0.04	0.10	0.19
24 or younger	91	1.60	5.92	0.03	0.10	0.18	0.29
25–29	561	1.74	10.47	0.02	0.07	0.12	0.19
30–34	417	0.82	5.90	0.00	0.03	0.09	0.18
35–39	174	0.61	5.97	0.00	0.02	0.05	0.13
40+	21	0.29	20.22	0.00	0.00	0.00	0.02
**TTP-Natural**							
		**Parameters**			**Fecundability**		
**Age (y) Group**	**n**	α	β	**q (Sterile)**	**First Quartile**	**Median**	**Third Quartile**
All ages	1264	1.88	10.73	0.14	0.05	0.11	0.19
24 or younger	91	1.72	6.35	0.04	0.10	0.18	0.29
25–29	561	2.58	15.08	0.06	0.07	0.13	0.19
30–34	417	1.75	9.40	0.22	0.02	0.10	0.19
35–39	174	1.31	8.64	0.30	0.00	0.06	0.15
40+	21	0.31	15.21	0.00	0.00	0.01	0.02

## Data Availability

Data may be available upon request to the corresponding author.

## Supplementary Materials

The following are available online at https://www.mdpi.com/article/10.3390/ijerph18105486/s1, Supplementary Table S1: Basic characteristics of the analytic sample by age category at the start of TTP shown as proportions (n = 1264).

## Appendix A. Detailed Description About the Maximum Likelihood Method

Fecundability and sterility by age was estimated using time to pregnancy (TTP) data and the maximum likelihood method. TTP can be written as (T,I), where T(0,1,2,…60) is the number of months between discontinuing contraception and either conceiving the first child (I=1) or censoring (I=0). Both TTP-all and TTP-natural were censored when a woman did not conceive (to give a birth) or was not currently pregnant at the time of survey. Additionally, TTP-natural was censored when a woman or her partner initiated fertility consultation or treatment. Descriptions below use the term TTP to refer to either type of TTP.

### Appendix A.1. One-Parameter Model

It is assumed that fecundability P(0≤P≤1) is constant for each woman and *P* is the same for all women in the same age group. The probability that a woman with fecundability *P* conceives at cycle T+1, i.e., being (T,1), is

$${\left(1-P\right)}^{T}P$$

The probability that a woman with fecundability *P* does not conceive at cycle T+1, i.e., (T,0), is

$${\left(1-P\right)}^{T+1}$$

### Appendix A.2. Two-Parameter Model

Assuming that fecundability *P* is constant for each woman and *P* follows a beta distribution, the probability density *f* of a woman having a fecundability *P* is

$$f\left(P\right)=\frac{\Gamma \left(\alpha +\beta \right)}{\Gamma \left(\alpha \right)\Gamma \left(\beta \right)}P^{\alpha -1}{\left(1-P\right)}^{\beta -1}\;\left(\alpha >0,\beta >0\right)$$

Thus, the probability of a woman being (T,1) is

$$\begin{array}{cc} \; & \int_{0}^{1}{\left(1-P\right)}^{T}Pf\left(P\right)dP\\ \; & =\int_{0}^{1}{\left(1-P\right)}^{T}P\frac{\Gamma (\alpha +\beta )}{\Gamma (\alpha )\Gamma (\beta )}P^{\alpha -1}{\left(1-P\right)}^{\beta -1}dP\\ \; & =\frac{\Gamma (\alpha +\beta )}{\Gamma (\alpha )\Gamma (\beta )}\int_{0}^{1}P^{\alpha }{\left(1-P\right)}^{\beta +T-1}dP\\ \; & =\frac{\Gamma (\alpha +\beta )}{\Gamma (\alpha )\Gamma (\beta )}\frac{\Gamma (\alpha +1)\Gamma (\beta +T)}{\Gamma (\alpha +\beta +T+1)}\\ \; & =\frac{\alpha \Gamma (\alpha +\beta )}{\Gamma (\beta )}\frac{\Gamma (\beta +T)}{\Gamma (\alpha +\beta +T+1)}=L\left(T\right) \end{array}$$

Similarly, the probability of a women being (T,0) is

$$\begin{array}{cc} \; & \int_{0}^{1}{\left(1-P\right)}^{T+1}f\left(P\right)dP\\ \; & =\int_{0}^{1}{\left(1-P\right)}^{T+1}\frac{\Gamma (\alpha +\beta )}{\Gamma (\alpha )\Gamma (\beta )}P^{\alpha -1}{\left(1-P\right)}^{\beta -1}dP\\ \; & =\frac{\Gamma (\alpha +\beta )}{\Gamma (\alpha )\Gamma (\beta )}\int_{0}^{1}P^{\alpha -1}{\left(1-P\right)}^{\beta +T}dP\\ \; & =\frac{\Gamma (\alpha +\beta )}{\Gamma (\alpha )\Gamma (\beta )}\frac{\Gamma (\alpha )\Gamma (\beta +T+1)}{\Gamma (\alpha +\beta +T+1)}\\ \; & =\frac{\Gamma (\alpha +\beta )}{\Gamma (\beta )}\frac{\Gamma (\beta +T+1)}{\Gamma (\alpha +\beta +T+1)}=L\left(T\right) \end{array}$$

The likelihood *L* can be calculated using the TTP data of *n* women as follows:

$$L=\prod_{i=1}^{n}L\left(T_{i}\right)$$

The maximum likelihood estimates of α and β are a set of those when *L* is at its maximum value. Logged *L* was used to find a set of parameters.

$$\log L=\log L\left(T_{1}\right)+\log L\left(T_{2}\right)+\ldots +\log L\left(T_{n}\right)$$

### Appendix A.3. Three-Parameter Model

We assumed that a proportion q(0≤q≤1) of women are sterile (=have zero probability of conception) and the rest have a fecundability *P* that follows a beta distribution. The probability density *f* of a woman having a fecundability *P* is

$$f\left(P\right)=q\delta_{0}+\left(1-q\right)\frac{\Gamma \left(\alpha +\beta \right)}{\Gamma \left(\alpha \right)\Gamma \left(\beta \right)}P^{\alpha -1}{\left(1-P\right)}^{\beta -1},$$

where $\delta_{0}$ is a delta distribution with a unit mass at P=0. The probability of a woman being (T,1) is

$$\begin{array}{cc} \; & \int_{0}^{1}{\left(1-P\right)}^{T}Pf\left(P\right)dP\\ \; & =\left(1-q\right)\int_{0}^{1}{\left(1-P\right)}^{T}P\frac{\Gamma (\alpha +\beta )}{\Gamma (\alpha )\Gamma (\beta )}P^{\alpha -1}{\left(1-P\right)}^{\beta -1}dP\\ \; & =\left(1-q\right)\frac{\Gamma (\alpha +\beta )}{\Gamma (\alpha )\Gamma (\beta )}\int_{0}^{1}P^{\alpha }{\left(1-P\right)}^{\beta +T-1}dP\\ \; & =\left(1-q\right)\frac{\Gamma (\alpha +\beta )}{\Gamma (\alpha )\Gamma (\beta )}\frac{\Gamma (\alpha +1)\Gamma (\beta +T)}{\Gamma (\alpha +\beta +T+1)}\\ \; & =\left(1-q\right)\frac{\alpha \Gamma (\alpha +\beta )}{\Gamma (\beta )}\frac{\Gamma (\beta +T)}{\Gamma (\alpha +\beta +T+1)}=L\left(T\right) \end{array}$$

The probability of a woman being (T,0) is

$$\begin{array}{cc} \; & \int_{0}^{1}{\left(1-P\right)}^{T+1}f\left(P\right)dP\\ \; & =q+\left(1-q\right)\int_{0}^{1}{\left(1-P\right)}^{T+1}\frac{\Gamma (\alpha +\beta )}{\Gamma (\alpha )\Gamma (\beta )}P^{\alpha -1}{\left(1-P\right)}^{\beta -1}dP\\ \; & =q+\left(1-q\right)\frac{\Gamma (\alpha +\beta )}{\Gamma (\alpha )\Gamma (\beta )}\int_{0}^{1}P^{\alpha -1}{\left(1-P\right)}^{\beta +T}dP\\ \; & =q+\left(1-q\right)\frac{\Gamma (\alpha +\beta )}{\Gamma (\alpha )\Gamma (\beta )}\frac{\Gamma (\alpha )\Gamma (\beta +T+1)}{\Gamma (\alpha +\beta +T+1)}\\ \; & =q+\left(1-q\right)\frac{\Gamma (\alpha +\beta )}{\Gamma (\beta )}\frac{\Gamma (\beta +T+1)}{\Gamma (\alpha +\beta +T+1)}=L\left(T\right) \end{array}$$

### Appendix A.4. Maximum Likelihood Estimate

A set of parameters (α and β for the two-parameter and α, β, and *q* for the three-parameter models) which yield the largest likelihood were obtained. Likelihood was calculated for every combination of the parameters with the rules described below.

#### Appendix A.4.1. Two-Parameter Models

First grid: Likelihood was calculated for 3000 sets of parameters, α ranged from 1 to 30 and β ranged from 1 to 100 with an increment of 1. When an estimated parameter with the maximum likelihood was the maximum of the set range, likelihood was calculated for wider ranges with an increment 1.

Second grid: Likelihood was calculated for narrower ranges which include the maximum likelihood estimates from the first grid, with an increment of 0.01 for α and 0.1 for β. When an estimated parameter with the maximum likelihood was either the minimum or the maximum of the set range, the likelihood calculations were repeated with a wider range with the same increment.

Third grid: Likelihood was calculated for ranges including the maximum likelihood estimates obtained from the second grid, with an increment of 0.01 for both α and β.

#### Appendix A.4.2. Three-Parameter Models

First grid: Likelihood was calculated for α ranged from 1 to 30 and β ranged from 1 to 50 with an increment of 1, and for *q* ranged from 0 to 0.99 with an increment of 0.01. When an estimated α or β with the maximum likelihood was the maximum of the set range, likelihood was calculated for wider ranges with an increment 1.

Second grid: Likelihood was calculated for narrower ranges which include the maximum likelihood estimates from the first grid, with an increment of 0.1 for both α and β. *q* ranged from 0 to 0.99 with an increment of 0.01. When an estimated α or β with the maximum likelihood was either the minimum or the maximum of the set range, the likelihood calculations were repeated with a wider range with the same increment.

Third grid: Likelihood was calculated for ranges including the maximum likelihood estimates obtained from the second grid, with an increment of 0.01 for both α and β, and 0.001 for *q*.

**Table A1:** Maximum log-likelihood and the mean (variance) of fecundability from one- and two-parameter models for all ages and by age group based on TTP-all or TTP-natural. *p*-values were obtained from chi-square distribution with one degree of freedom.

TTP-All					
		**Fecundability, Mean (Variance)**			
**Age (y) Group**	**n**	**Two-Parameter Models**	**One-Parameter Models**		
All	1264	0.13 (0.01)	0.07 (0.00)		
24 or younger	91	0.21 (0.03)	0.11 (0.00)		
25–29	561	0.14 (0.01)	0.09 (0.00)		
30–34	417	0.12 (0.01)	0.06 (0.00)		
35–39	174	0.09 (0.01)	0.04 (0.00)		
40+	21	0.02 (0.00)	0.01 (0.00)		
		**Maximum Log-Likelihood**			
**Age (y) Group**	**n**	**Two-Parameter Models (a)**	**One-Parameter Models (b)**	**2{(a)–(b)}**	***p*** **-Value**
All	1264	−3350.67	−3498.01	294.69	0.000
24 or younger	91	−239.40	−252.39	25.98	0.000
25–29	561	−1507.31	−1555.25	95.88	0.000
30–34	417	−1111.52	−1162.00	100.96	0.000
35–39	174	−427.49	−444.62	34.26	0.000
40+	21	−22.62	−22.73	0.21	0.646
**TTP-Natural**					
		**Fecundability, Mean (Variance)**			
**Age (y) Group**	**n**	**Two-Parameter Models**	**One-Parameter Models**		
All	1264	0.13 (0.02)	0.07 (0.00)		
24 or younger	91	0.21 (0.02)	0.12 (0.00)		
25–29	561	0.14 (0.01)	0.09 (0.00)		
30–34	417	0.13 (0.02)	0.06 (0.00)		
35–39	174	0.10 (0.02)	0.04 (0.00)		
40+	21	0.02 (0.00)	0.01 (0.00)		
		**Maximum Log-Likelihood**			
**Age (y) Group**	**n**	**Two-Parameter Models (c)**	**One-Parameter Models (d)**	**2{(c)–(d)}**	***p*** **-Value**
All	1264	−2870.59	−3000.66	260.14	0.000
24 or younger	91	−234.63	−246.43	23.61	0.000
25–29	561	−1346.07	−1382.87	73.60	0.000
30–34	417	−907.64	−966.84	118.39	0.000
35–39	174	−323.91	−339.84	31.86	0.000
40+	21	−15.67	−15.70	0.06	0.813

**Table A2:** Maximum log-likelihood of two- and three-parameter models by age group, based on TTP-all or TTP-natural. *p*-values were obtained from chi-square distribution with one degree of freedom.

TTP-All				
	**Maximum Log-Likelihood**			
**Age (y) Group**	**Three-Parameter Models (a)**	**Two-Parameter Models (b)**	**2{(a)–(b)}**	***p*** **-Value**
All	−3350.60	−3350.67	0.14	0.710
24 or younger	−239.30	−239.40	0.21	0.648
25–29	−1507.04	−1507.31	0.54	0.463
30–34	−1111.52	−1111.52	−0.01	1.000
35–39	−427.49	−427.49	0.00	1.000
40+	−22.63	−22.62	0.00	1.000
**TTP-Natural**				
	**Maximum Log-Likelihood**			
**Age (y) Group**	**Three-Parameter Models (c)**	**Two-Parameter Models (d)**	**2{(c)–(d)}**	***p*** **-Value**
All	−2865.78	−2870.59	9.64	0.002
24 or younger	−234.51	−234.63	0.24	0.625
25–29	−1344.58	−1346.07	2.99	0.084
30–34	−904.84	−907.64	5.61	0.018
35–39	−323.55	−323.91	0.72	0.398
40+	−15.67	−15.67	0.00	1.000

**Table A3:** Estimated parameters of the two-parameter models and the median, the first and third quartiles, and the mean and variance of fecundability estimated using TTP-natural for all ages and by age group. The analytic sample was limited to those who discontinued contraception within 24 months before the survey (n = 525).

		Parameters		Fecundability				
**Age (y) Group**	**n**	α	β	**First Quartile**	**Median**	**Third Quartile**	**Mean**	**Variance**
All	525	0.82	4.87	0.04	0.10	0.21	0.14	0.02
24 or younger	31	0.93	2.16	0.11	0.25	0.45	0.30	0.05
25–29	223	1.65	10.25	0.06	0.12	0.19	0.14	0.01
30–34	183	0.77	4.44	0.04	0.10	0.22	0.15	0.02
35–39	76	0.26	1.87	0.00	0.03	0.17	0.12	0.03
40+	12	1.19	147.43	0.00	0.01	0.01	0.01	0.00

## References

[1] Bongaarts J., Potter R.G. (1983). Fertility, Biology, and Behavior. An Analysis of the Proximate Determinants.

[2] Wood J.W. (1994). Dynamics of Human Reproduction.

[3] Gini C. (1926). Decline in the birth-rate and the “fecundability” of woman. Eugen. Rev..

[4] Bendel J.P., Hua C. (1978). An estimate of the natural fecundability ratio curve. Soc. Biol..

[5] Schwartz D., Mayauz M.J. (1982). Female fecundity as a function of age. N. Engl. J. Med..

[6] Rothman K.J., Wise L.A., Sørensen H.T., Riis A.H., Mikkelsen E.M., Hatch E.E. (2013). Volitional determinants and age-related decline in fecundability: A general population prospective cohort study in Denmark. Fertil. Steril..

[7] Konishi S., Sakata S., Oba S.M., O’Connor K.A. (2018). Age and time to pregnancy for the first child among couples in Japan. J. Popul. Stud..

[8] Weinberg C.R., Gladen B.C. (1986). The Beta-Geometric Distribution Applied to Comparative Fecundability Studies. Biometrics.

[9] Wood J.W., Holman D.J., Yashin A.I., Peterson R.J., Weinstein M., Chang M.-C. (1994). A Multistate Model of Fecundability and Sterility. Demography.

[10] Dunson D.B., Baird D.D., Colombo B. (2004). Increased infertility with age in men and women. Obstet. Gynecol..

[11] Ecochard R. (2006). Heterogeneity in fecundability studies: Issues and modelling. Stat. Methods Med. Res..

[12] Dyer S., Chambers G.M., de Mouzon J., Nygren K.G., Zegers-Hochschild F., Mansour R., Ishihara O., Banker M., Adamson G.D. (2016). International Committee for Monitoring Assisted Reproductive Technologies world report: Assisted Reproductive Technology 2008, 2009 and 2010. Hum. Reprod..

[13] Steiner A.Z., Jukic A.M.Z. (2016). Impact of female age and nulligravidity on fecundity in an older reproductive age cohort. Fertil. Steril..

[14] Bianchi S., Macchiarelli G., Micara G., Linari A., Boninsegna C., Aragona C., Rossi G., Cecconi S., Nottola S.A. (2015). Ultrastructural markers of quality are impaired in human metaphase II aged oocytes: A comparison between reproductive and in vitro aging. J. Assist. Reprod. Genet..

[15] Navot D., Bergh R.A., Williams M.A., Garrisi G.J., Guzman I., Sandler B., Grunfeld L. (1991). Poor oocyte quality rather than implantation failure as a cause of age-related decline in female fertility. Lancet.

[16] Curtis K.M., Savitz D.A., Arbuckle T.E. (1997). Effects of Cigarette Smoking, Caffeine Consumption, and Alcohol Intake on Fecundability. Am. J. Epidemiol..

[17] O’Connor K.A., Holman D.J., Wood J.W. (1998). Declining fecundity and ovarian ageing in natural fertility populations. Maturitas.

[18] McKinnon C.J., Hatch E.E., Rothman K.J., Mikkelsen E.M., Wesselink A.K., Hahn K.A., Wise L.A. (2016). Body mass index, physical activity and fecundability in a North American preconception cohort study. Fertil. Steril..

[19] Wesselink A.K., Hatch E.E., Rothman K.J., Mikkelsen E.M., Aschengrau A., Wise L.A. (2019). Prospective study of cigarette smoking and fecundability. Hum. Reprod..

[20] Willis S.K., Hatch E.E., Wesselink A.K., Rothman K.J., Mikkelsen E.M., Wise L.A. (2019). Female sleep patterns, shift work, and fecundability in a North American preconception cohort study. Fertil. Steril..

[21] Van Eekelen R., Scholten I., Tjon-Kon-Fat R.I., Van Der Steeg J.W., Steures P., Hompes P., Van Wely M., Van Der Veen F., Mol B.W., Eijkemans M.J. (2017). Natural conception: Repeated predictions overtime. Hum. Reprod..

[22] McLernon D.J., Lee A.J., Maheshwari A., Van Eekelen R., Van Geloven N., Putter H., Eijkemans M.J., Van Der Steeg J.W., Van Der Veen F., Steyerberg E.W. (2019). Predicting the chances of having a baby with or without treatment at different time points in couples with unexplained subfertility. Hum. Reprod..

[23] Bianchi S., Nottola S.A., Torge D., Palmerini M.G., Necozione S., Macchiarelli G. (2020). Association between Female Reproductive Health and Mancozeb: Systematic Review of Experimental Models. Int. J. Environ. Res. Public Health.

[24] Buck Louis G.M., Sundaram R., Schisterman E.F., Sweeney A.M., Lynch C.D., Gore-Langton R.E., Chen Z., Kim S., Caldwell K.L., Barr D.B. (2012). Heavy metals and couple fecundity, the LIFE Study. Chemosphere.

[25] Buck Louis G.M., Sundaram R., Sweeney A.M., Schisterman E.F., Maisog J., Kannan K. (2014). Urinary bisphenol A, phthalates, and couple fecundity: The Longitudinal Investigation of Fertility and the Environment (LIFE) Study. Fertil. Steril..

[26] Belli M., Palmerini M.G., Bianchi S., Bernardi S., Khalili M.A., Nottola S.A., Macchiarelli G. (2021). Ultrastructure of mitochondria of human oocytes in different clinical conditions during assisted reproduction. Arch. Biochem. Biophys..

