# Supplementary material for: Fecundability and Sterility by Age: Estimates Using Time to Pregnancy Data of Japanese Couples Trying to Conceive Their First Child with and without Fertility Treatment
Source: Int J Environ Res Public Health. 2021 May 20;18(10):5486. doi: 10.3390/ijerph18105486 (PMC8161326; doi:10.3390/ijerph18105486)
Supplement: Supplementary file 1 [file ijerph-18-05486-s001.zip › ijerph-1203684-SI.pdf]

Supplementary Table S1. Basic characteristics of the analytic sample by age category at the start of TTP shown as proportions (%) (n=1264).

	Total	Age (year) at the start of TTP				
		24 or younger (n=91)	25-29 (n=561)	30-34 (n=417)	35-39 (n=174)	40+ (n=21)
All	100	100	100	100	100	100
Partnership status						
No partner	1	0	1	0	1	0
Partnered, not engaged	1	1	1	1	1	10
Partnered, engaged	2	2	2	2	3	10
Married	95	95	95	96	94	76
Widowed	0	0	0	0	0	0
Separated or divorced	1	2	2	1	1	5
Eligibility group						
Parous (group A)	62	78	70	56	52	14
Nulliparous and currently pregnant (group B)	13	11	12	17	10	5
Nulliparous, not currently pregnant, and at risk of pregnancy (group C)	25	11	19	27	39	81
Age difference between participants and their partner						
Partner younger	21	12	12	27	40	52
Partner older by 0-9 years	72	75	82	66	56	43
Partner older by 10+ years	7	13	6	6	5	5

Parity						
0	38	22	30	44	48	86
1	49	52	53	47	47	14
2	13	25	16	9	5	0
3	0	1	1	0	0	0
4	0	0	0	0	0	0
5	0	0	0	0	0	0
Spontaneous abortion[a]						
Never	87	91	90	86	79	62
Ever	13	9	10	14	21	38
Stillbirth[a]						
Never	99	100	100	99	98	100
Ever	1	0	0	1	2	0
Induced abortion[a]						
Never	92	93	93	93	92	81
Ever	8	7	7	7	8	19
Educational background						
Junior high school	1	2	1	1	1	0
Senior high school	17	42	14	16	17	10
Junior college	18	21	18	16	18	33
College	15	9	14	14	21	38
University	44	25	48	47	38	19
Graduate	5	0	5	6	5	0
Other	0	1	0	0	1	0
Last method of contraception (multiple answers)						
Condom	81	87	81	82	75	62
Oral contraceptives	7	2	9	6	6	5

Morning pill	0	0	0	0	0	0
Withdrawal	19	22	16	20	22	43
Calendar method	5	5	5	5	4	0
BBT method	8	4	8	9	9	10
Other contraceptive method	1	0	0	1	1	0
Infertility treatment						
Ever	18	5	12	23	29	52
Never	82	95	88	77	71	48
Methods of infertility treatment						
Timing method	16	5	12	21	24	48
Ovulation induction	10	3	8	12	12	33
Artificial insemination	6	0	3	7	11	29
In vitro fertilization	3	0	1	3	9	29
Micro-insemination	3	0	1	3	9	19
Other	0	0	0	0	0	5

a Before the first birth for women in group A.

TTP: time to pregnancy
